# Tauber’s antegrade sclerotherapy for the treatment of varicocele in children and adolescents. Does the pubertal stage matter?

**DOI:** 10.1007/s00345-025-05564-x

**Published:** 2025-03-19

**Authors:** Carlotta Plessi, Nino Guarino, Gabriele Vasta, Vito Briganti, Guido Fiocca

**Affiliations:** 1 Pediatric Surgery Unit, Department of Medical, Surgical and Neurological Sciences, S. Maria alle Scotte Hospital, University of Siena, Siena, Italy; 2 Pediatric Surgery Unit, San Camillo-Forlanini Hospital, C.ne Gianicolense, 87, Rome, 00152 Italy

**Keywords:** Varicocele, Children, Adolescents, Antegrade sclerotherapy, Pubertal stage

## Abstract

**Purpose:**

To compare the results of Tauber’s antegrade sclerotherapy at different pubertal stages and, notably, to evaluate its feasibility in pre-pubertal age.

**Methods:**

We retrospectively reviewed the clinical records of patients that underwent antegrade sclerotherapy at our centre between 2005 and 2019. We divided our population into groups based on pubertal stage according to Tanner’s classification. Pre-operative, intra-operative and post-operative data were collected. Finally, we assessed the association between Tanner’s stage at surgery, operative characteristics, short-term complications and long-term outcomes.

**Results:**

513 patients were included in our study. Median age was 13 years (range 9–17). 467 (91%) underwent the procedure under local anaesthesia or with mild sedation. Median operative time was 23 min. In 7 cases (1%) conversion was necessary due to intraoperative complications. In 31 cases (6%) we observed minor post-operative complications. Recurrence rate was 3%. Data analyses revealed that there were statistically significant differences among different Tanner’s pubertal stages at the intervention in terms of type of anaesthesia (*p* = 0.009). No difference was found in terms of operative time (*p* = 0.214), conversion’s rate (*p* = 0.406), postoperative complications (*p* = 0.159), and clinical outcomes at 1 month (*p* = 0.916), 6 month (*p* = 0.112) and at final follow up (*p* = 0.312).

**Conclusion:**

Tauber’s antegrade sclerotherapy is a reliable technique in the treatment of paediatric and adolescent varicocele, regardless of patients’ pubertal stage. Although prepubertal patients may need more general anaesthesia, the procedure maintains similar efficacy and complication rates across all age groups.

## Introduction

Varicocele is one of the most frequent urogenital disorders affecting adolescent males, with an incidence of approximately 15%. Despite this, it remains one of the most controversial issues in paediatric urology. The main topics of discussion concern the indications for surgical treatment, the effect on future fertility, and finally the choice of surgical technique. In fact, while in adulthood paternity failure is a clear indication for surgery and a primary outcome for clinical trials, in paediatric age it is more difficult to find a valid endpoint and we must consider as criteria for surgical intervention some indirect indicators of testicular damage, mainly testicular dimensions and sperm analyses [1, 2]. There is currently no definitive evidence that the treatment at an earlier age improves the prognosis in terms of fertility [3]. However, the surgical treatment of varicocele has shown to improve sperm characteristics and to result in the so-called “testicular catch-up growth” [4]. Moreover, in the adult population, testicular damage has been proven to be time-related [5].

Numerous studies have described various surgical techniques, including different types of open varicocelectomy and minimally invasive techniques, such as microsurgical, laparoscopic or robotic varicocelectomy [6, 7]. Anyway, there is no consensus on which of these techniques should be considered the best. Tauber’s antegrade sclerotherapy has demonstrated to have success rates comparable to other techniques, together with lower complications’ rate particularly in terms of postoperative hydroceles [8]. It also has clear advantages in terms of cost-effectiveness: it can be performed under local anaesthesia, requires few instruments, short surgical time, and hospitalization time [9]. The population affected by varicocele in paediatric and adolescent age is extremely variable, as it ranges across different ages and most importantly different Tanner’s stages. Despite this, there are no studies comparing the results of Tauber’s antegrade sclerotherapy at different pubertal stages and, notably, there are no studies showing the results of this technique at pre-pubertal age (Tanner stage I). Based on the hypothesis that testicular damage from varicocele may be time-related and that early intervention could be beneficial, our study aims to compare operative characteristics, short-term complications and outcomes of Tauber’s antegrade sclerotherapy across different pubertal stages. Additionally, we seek to evaluate the feasibility and potential benefits of this technique in pre-pubertal patients.

## Methods

### Study design

We performed a retrospective observational study including all patients that underwent antegrade sclerotherapy in our centre between 2005 and 2019. The inclusion criteria were an antegrade sclerotherapy for varicocele and age $\:<$18 years at the intervention. The exclusion criteria were a previous intervention for varicocele (recurrence). Minimal postoperative follow-up was six months.

### Clinical management and surgical technique

At our clinic, surgery is indicated for patients diagnosed with varicocele in case of testicular asymmetry (i.e., percentage volume difference between the two testicles > 20%), symptoms (primarily testicular pain), bilateral varicocele or left varicocele with contralateral previous testicular or inguinal surgery. Moreover, the presence of severe varicocele according to Dubin et Amelar ‘s [10] and Hirsh’s [11] classifications (i.e., Dubin grade 3 and Hirsh grade 3) was an indication for surgical treatment [12].

At our centre, we chose Tauber’s antegrade sclerotherapy [13] as the primary approach for treating paediatric and adolescent varicocele. Each patient for whom a surgical indication is given is offered the procedure under local anesthesia. General anesthesia is only utilized if the patients or their family refuse this option.

At the beginning of the procedure the patient is positioned supine, in a slight anti-Trendelenburg tilt, ensuring a correct exposure to the X-ray tube. After disinfection and draping of the operative field, a 2 cm transverse incision is made at the base of the scrotum, over the spermatic chord, approximately 2 cm under the external inguinal ring. The subcutaneous layer is incised, and the spermatic chord mobilized and grasped with an Ellis forceps. Next, the spermatic fasciae are incised to expose the pampiniform plexus. The most dilated vein within the plexus is isolated, and a Vicryl loop is placed both proximally and distally the segment designated for phlebotomy. Following proximal ligation, phlebotomy is performed, and a 20-gauge cannula for peripheral vascular access is inserted into the vein and fixed with the second Vycril loop, taking care of not occluding the cannula. If catheter placement is unsuccessful, the vessel is ligated, and the process can be repeated on an alternative vein. A first phlebography is performed to confirm the drainage in the internal spermatic vein, then sclerosis is performed using 1–2 cc of sclerotising agent in foam form. The type of agent varied in different periods: in a first phase tetradecyl sulfate sodium salt (Fibrovein^®^ 3% or Trombovar^®^ 3%) was used, then it was substituted by polidocanol (Atoxisclerol^®^ 3%). During the injection phase, the contrast medium is used as guide to control the progression of the sclerotizing agent, and the injection continues until fluoroscopy confirms its complete disappearance. During this injection patients are instructed to perform the Valsalva maneuver. Finally, the cannulated vein is ligated on both sides of the phlebotomy site, and the skin and subcutaneous layers are sutured.

Follow-up consisted in physical examination, testicular volume assessment with Prader orchydometer and doppler assessment of venous reflux in the spermatic cord at 1 week, 1 month and 6 months after surgery, then continued annually until complete pubertal development. Doppler ultrasounds and sperm analyses are not systematically performed at our centre.

### Data collection

First, we clustered our population into groups, basing on the Tanner’s pubertal stage (from stage I to stage V).

Pre-operative (family history and surgical indication), intraoperative (operative time, type of anaesthesia, conversion to other surgery) and postoperative (short-term complications, 1-month clinical outcome, 6-month clinical outcome, final clinical outcome, and catch-up growth) data were systematically collected from patients’ hospital files.

Regarding the follow up, the final clinical outcome was meant as the outcome at Tanner stage V for all patients operated at Tanner stage I-IV. For patients operated at Tanner stage V it was considered the outcome at 1-year postoperative follow-up.

Clinical outcome at one-month, clinical outcome at six months and final outcome were considered as dichotomic variables and classified as “resolution” (in case of varicocele resolution or reduction to a Hirsch grade 1) or “recurrence” (in case of persistence of Hirsch grade 2–3 or symptomatic varicocele). Catch-up growth was also analysed for patients that presented with a pre-operative testicular asymmetry: it was considered as a dichotomic variable and classified as “present” or “absent”.

### Data analysis

During the data analysis, categorical data were described by absolute frequency and relative frequency (in percentage), continuous data by median and range. Regarding operative time, we excluded from the analyses data from patients that underwent other procedures at the same time. A one-way ANOVA was used to determine whether there was a statistically significant difference between different Tanner’s pubertal stages (I-V). A Chi-Square Test was performed to assess the relationship between Tanner’s pubertal stage (I-V) at the intervention and all the other categorical variables. Significance was fixed at *p* = 0.05 and all analyses were carried out with IBM SPSS Statistics for Windows version 25 (IBM Corp, Armonk, NY, USA).

## Results

Between 2005 and 2019, 772 paediatric patients (age < 18 years) were treated for varicocele with antegrade sclerotherapy at our centre. Twelve patients were excluded basing on the exclusion criteria (recurrence), 247 were excluded because they didn’t reach the minimal postoperative follow-up. Eventually, 513 patients were included in the present study. Median age was 13 years (range 9–17). We clustered them into five groups, based on Tanner pubertal stage at surgery: 103 cases (20%) were Tanner I, 82 (16%) Tanner II, 162 (32%) Tanner III, 71 (14%) Tanner IV, 95 (19%) Tanner V.

Patients’ characteristics, including indications to treatment, are shown in Table 1. 108 patients (21%) had family history of varicocele, 107 patients (21%) of other varicose pathologies. 472 patients (92%) had a Hirsh grade 3 at the intervention. 498 patients (97%) had a dubin grade 3 at the intervention. 39 patients (8%) had symptoms (i.e., testicular pain), 87 (17%) had testicular asymmetry, 18 (4%) had bilateral varicocele, 16 (3%) had a simultaneous contralateral inguinal or testicular affection.

**Table 1:** Patients’ characteristics clustered for Tanner pubertal stage at surgery

Tanner’s stage		I (*n* = *103*)	II (*n* = *82*)	III (*n* = *162*)	IV (*n* = *71*)	V (*n* = *95*)	All (*n* = *513*)
*Median age in years (range)*		12 (9–14)	13 (11–16)	13 (11–17)	14 (12–17)	16 (12–17)	13 (9–17)
Family history	*Varicocele*	23 (22%)	19 (23%)	30 (18%)	16 (23%)	20 (21%)	108 (21%)
	*Varicose veins*	16 (16%)	19 (23%)	32 (20%)	21 (30%)	17 (18%)	105 (20%)
	*Haemorroids*	1 (1%)	0 (0%)	1 (1%)	0 (0%)	0 (0%)	2 (1%)
	*Nothing*	63 (61%)	44 (54%)	99 (61%)	34 (48%)	58 (61%)	298 (58%)
Surgical indications	*Dubin 3 and Hirsch 3*	95 (92%)	78 (95%)	152 (94%)	61 (86%)	81 (85%)	467 (91%)
	*Symptoms*	7 (7%)	4 (5%)	12 (7%)	8 (11%)	8 (8%)	39 (8%)
	*Asymmetry*	14 (14%)	5 (6%)	21 (13%)	19 (27%)	28 (29%)	87 (17%)
	*Bilaterality*	5 (5%)	3 (4%)	4 (2%)	2 (3%)	4 (4%)	18 (4%)
	*Contralateral pathology*	1 (1%)	1 (1%)	9 (6%)	1 (1%)	4 (4%)	16 (3%)

**Table 2:** Operative data clustered for Tanner pubertal stage at surgery

Tanner’s stage		I (*n* = *103*)	II (*n* = *82*)	III (*n* = *162*)	IV (*n* = *71*)	V (*n* = *95*)	All (*n* = *513*)
*Median operative time in minutes (range)*		24 (15–122)	22 (15–61)	23 (15–64)	24 (15–64)	23 (15–67)	23 (15–122)
*Anaesthesia*	*Local*	81 (79%)	73 (89%)	131 (81%)	59 (83%)	84 (89%)	428 (83%)
	*Local with sedation*	6 (6%)	4 (5%)	17 (10%)	5 (7%)	7 (7%)	39 (8%)
	*General*	16 (15%)	5 (6%)	14 (9%)	7 (10%)	4 (4%)	46 (9%)
*Conversion to other surgery*		2 (2%)	1 (1%)	0 (0%)	2 (3%)	2 (2%)	7 (1%)

Operative data are shown in Table 2. Median operative time was 23 min (range 15–122). A one-way ANOVA revealed that there was no significant difference in terms of operative time (*p* = 0.214) between different pubertal stages. Anaesthesia was local in 428 cases (83%), local with sedation in 39 (8%) and general in 46 (9%). In pre-pubertal age (Tanner stage I) general anaesthesia was necessary in the 15% of the cases, while in Tanner’s stage II-V in was necessary in the 7%. The Chi Square test showed a significant association between Tanner stage I at the intervention and the need of general anaesthesia (X2 = 6.809, *p* = 0.009). In 7 cases (1%) there was the need of a conversion to another technique, because of technical difficulties or anatomical anomalies. The Chi Square test revealed that there was no significant relationship between Tanner’s pubertal stage at the intervention and conversion rates (X2 = 3.999, *p* = 0.406).

**Table 3:** Patients’ outcomes clustered for Tanner pubertal stage at surgery

Tanner’s stage		I (*n* = *103*)	II (*n* = *82*)	III (*n* = *162*)	IV (*n* = *71*)	V (*n* = *95*)	All (*n* = *513*)
Short-term complications	*Hematoma*	1 (1%)	2 (2%)	2 (1%)	3 (4%)	5 (5%)	13 (3%)
	*Wound dehiscence*	1 (1%)	2 (2%)	5 (3%)	1 (1%)	1 (1%)	10 (2%)
	*Oedema*	1 (1%)	0 (0%)	0 (0%)	0 (0%)	1 (1%)	2 (1%)
	*Transient hydrocele*	0 (0%)	0 (0%)	0 (0%)	2 (3%)	2 (2%)	4 (1%)
	*Persistent pain*	0 (0%)	0 (0%)	0 (0%)	0 (0%)	2 (2%)	2 (1%)
	*Total*	3 (3%)	4 (5%)	7 (4%)	6 (8%)	11 (12%)	31 (6%)
Outcome at 1 month	*Resolution*	99 (96%)	80 (98%)	158 (98%)	70 (99%)	94 (99%)	501 (98%)
	*Recurrence*	4 (4%)	2 (2%)	4 (2%)	1 (1%)	1 (1%)	12 (2%)
Outcome at 6 months	*Resolution*	99 (96%)	78 (95%)	157 (97%)	70 (99%)	93 (98%)	492 (96%)
	*Recurrence*	4 (4%)	4 (5%)	5 (3%)	1 (2%)	2 (2%)	19 (4%)
Final clinical outcome	*Resolution*	57 (55%)	53 (65%)	136 (84%)	60 (84%)	90 (95%)	396 (77%)
	*Recurrence*	4 (4%)	3 (3%)	3 (2%)	2 (3%)	2 (2%)	14 (3%)
	*Lost to follow up*	42 (41%)	26 (32%)	23 (14%)	9 (13%)	3 (3%)	103 (20%)

**Fig. 1:**
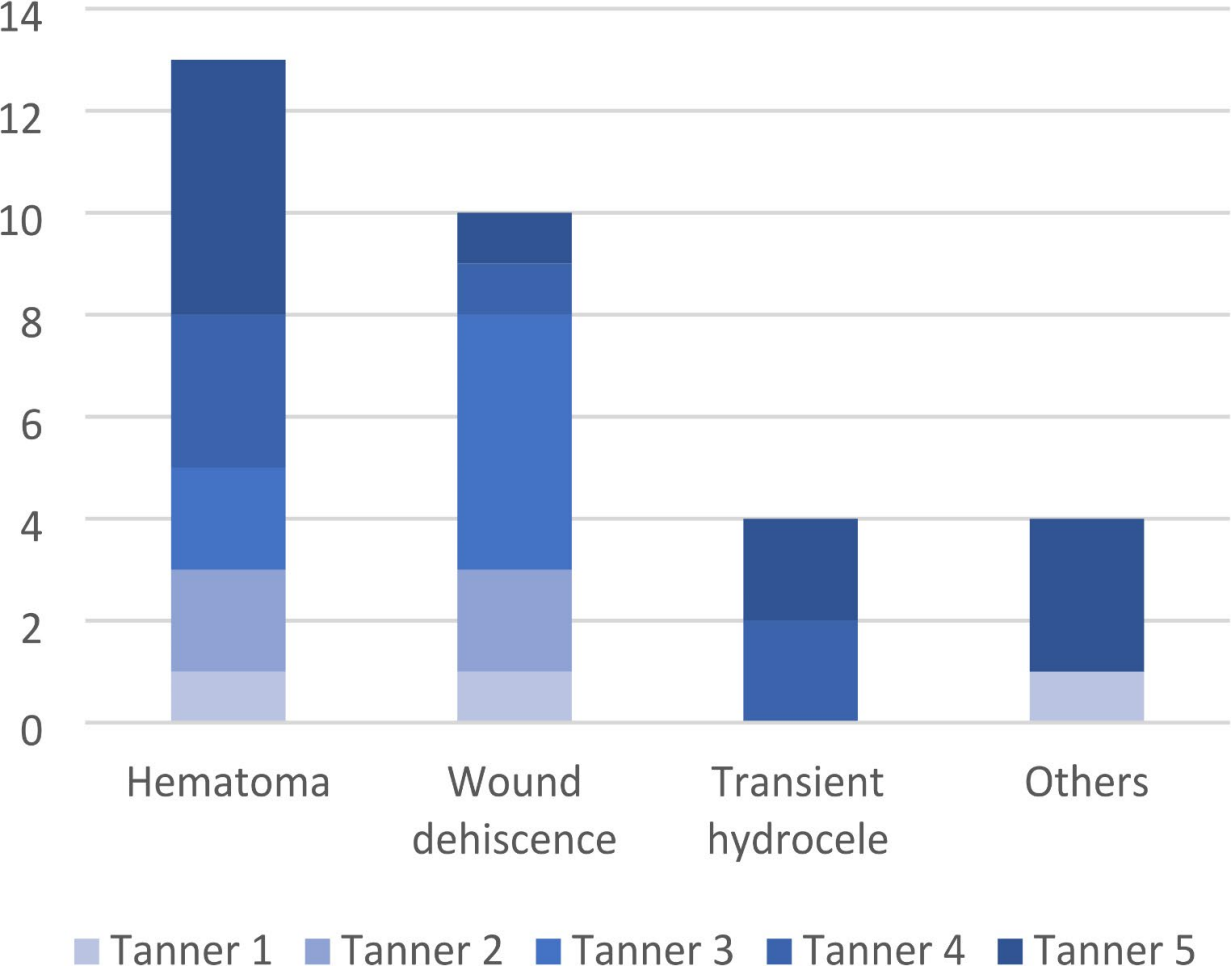
Short-term postoperative complications clustered by Tanner’s pubertal stage

Data regarding patients’ follow-up are shown in Table 3. A total of 31 short-term complications (< 30 postoperative days) were observed, with an overall rate of 6%. All of them were minor complications (grade I in the Clavien-Dindo classification). Their distribution by Tanner’s pubertal stage is shown in Fig. 1. The most frequent short-term complication was hematoma (42% of total complications). The Chi Square test revealed that there was no significant relationship between Tanner’s pubertal stage at the intervention and complications (X2 = 1.979, *p* = 0.159). A complete follow-up was reached in 410 patients (80%) and, more specifically, by the 59% of patients operated at Tanner stage I, 68% of patients operated at stage II, 86% of patients operated at stage III, 87% of patients operated at stage IV and 97% of patients operated at stage V. Of these 410 patients with a complete follow up, 14 (3%) showed a recurrence of varicocele at the end of the follow up. The Chi Square test revealed that there was no significant relationship between Tanner’s pubertal stages at the intervention and clinical outcome at 1 month (X2 = 0.956, *p* = 0.916), 6 month (X2 = 7.499, *p* = 0.112) and at the end of the follow up (X2 = 4.764, *p* = 0.312). Finally, between 87 patients presenting with a significant testicular asymmetry before the intervention, 67 (77%) presented an effective catch-up growth in the postoperative follow up, more precisely 14 (100%) in the pre-puberal group and 53 (73%) in the other groups. However, no statistical difference was found in terms of catch-up growth between patients at different Tanner’s pubertal stage at the intervention (X2 = 8.926, *p* = 0.06).

## Discussion

The indications for surgical treatment of varicocele in paediatric and adolescent age are still a debated issue, mainly due to the difficulties in proving a correlation with impaired fertility and paternity rates later in adulthood. Indirect signs of impaired testicular health have been recognised in patients affected by varicocele in paediatric age (mainly testicular volume and sperm analyses in older patients) and a beneficial effect of surgical treatment has been demonstrated, in terms of testicular catch-up growth and improvement in sperm parameter in older patients [14]. However, there is limited evidence supporting the benefits of early treatment, especially in prepubertal patients, where the long-term fertility outcomes remain uncertain [3]. As such, the ethical and practical challenges of performing elective surgeries on very young patients with unclear long-term fertility benefits further complicate decision-making in this area. Moreover, there is a lack of literature comparing the outcomes of varicocele treatment across different pubertal stages.

At our centre, Tauber’s antegrade sclerotherapy is the treatment of choice for adolescent varicocele. Since its description in 1994 [13], this technique has demonstrated to be feasible and efficient in treating varicocele, also in paediatric population [15]. However, it is undeniable that it can present some critical points, especially in the paediatric population. In fact, the veins of the pampiniform plexus may be small and difficult to canulate: this may increase the operating time, especially in the case of a limited expertise of the surgeon, increase the risk of intra-operative complications other than hydrocele (e.g., scrotal haematoma) and increase the risk of conversion to another technique [8]. In our study, mean operative time was only 23 min, that is quite short compared to previous studies [8, 16]. Conversion to another technique was necessary in 1% of the procedures, a rate consistent with findings from a recent systematic review and metanalysis by Tandon et al. [17]. The authors also noted that the increased risk of technical failure associated with sclerosing techniques is particularly common with the retrograde approach. Regarding post-operative complications, the overall rate in our study reached the 6%, the most frequent represented by scrotal hematoma (3%), while postoperative transient hydrocele occurred in only 4 patients (1%). This is consistent with the results of Tandon’s metanalysis [17], that found a low incidence of postoperative hydrocele (between 1 and 4%) but a relatively higher rate of other peri-operative complications (between 0 and 6%) compared to other techniques. To gain a deeper insight into the topic, we compared the feasibility of the intervention across different pubertal stages. Our analysis revealed no significant differences in the duration of the intervention, the need for conversion, or post-operative complications between the groups. Another problem with Tauber’s technique in paediatric age is that to be performed under local anaesthesia it requires a certain degree of cooperation from the patient. Nevertheless, in 91% of our patients, we managed to avoid general anaesthesia, with (8%) or without (83%) the need of bland sedation. Interestingly, we observed a significant association between prepubertal Tanner’s stage and the need of general anaesthesia. This can be easily explained by the fact that younger patients may be uncomfortable undergoing the procedure while awake under local anaesthesia. Regarding recurrences, the aforementioned metanalysis [17] reported an overall rate of 7.6%, which was higher compared to other techniques. However, it is important to note that comparing recurrence rates between different studies and techniques can be challenging. As mentioned by Locke et al. in their review in 2017 [4], varicocele’s recurrence rate in different studies is widely variable (0–31%), probably due to variable definitions and different follow-up systems (clinical evaluation or Doppler ultrasound). In our study, recurrence was defined as the persistence of Hirsch grade 2–3 or symptomatic varicocele at follow-up clinical examinations. Moreover, for the patients presenting with significant (> 20%) testicular asymmetry pre-operatively, we evaluated the catch-up growth. Recurrence rate in our population was low (3%) and catch-up growth in the sub-group presenting with testicular asymmetry was 77%, in line with previous series [17]. Again, we compared our results between different pubertal stages, and we found no differences in terms of recurrence rate, demonstrating the efficacy of this technique in paediatric age regardless of pubertal stage. However, despite patients operated at a pre-pubertal stage showed a higher rate of catch-up growth (100% vs. 73%) the difference was not statistically significant.

Although the present study presents many interesting results, it is appropriate to recognize several potential limitations. First, its retrospective design limits the generalizability of the findings. Second, systematic sperm analysis and ultrasonographic evaluations, which are useful tools for assessing the effect of varicocele on gonadal function, could have enhanced interobserver reliability and methodological rigor. Third, the high rate of lost to follow-up, particularly in the lower pubertal stages, may have influenced the results.

In conclusion, Tauber’s antegrade sclerotherapy is a feasible and effective technique for treatment of adolescent varicocele, regardless of patient’s pubertal stage at the intervention. However, the need for general anaesthesia was found to be increased in pre-pubertal patients. Further prospective studies, with more structured follow up, are needed to confirm our results and to validate the use of this technique in prepubertal patients.

## Data Availability

All data supporting the findings of this study are available from the corresponding author on reasonable request.
